# Racial differences in B cell receptor signaling pathway activation

**DOI:** 10.1186/1479-5876-10-113

**Published:** 2012-06-06

**Authors:** Diane M Longo, Brent Louie, Kavita Mathi, Zoltan Pos, Ena Wang, Rachael E Hawtin, Francesco M Marincola, Alessandra Cesano

**Affiliations:** 1 Nodality, South San Francisco, CA 94080, USA; 2 MedImmune, Mountain View, CA 94043, USA; 3 Department of Genetics, Cell and Immunobiology, Semmelweis University, Budapest, H-1089, Hungary; 4 Infectious Disease and Immunogenetics Section, Department of Transfusion Medicine, Clinical Center, and Center for Human Immunology, National Institutes of Health, Bethesda, MD 20892, USA

**Keywords:** Multi-parameter flow cytometry, BCR signaling, Race

## Abstract

**Background:**

Single-cell network profiling (SCNP) is a multi-parametric flow cytometry-based approach that simultaneously measures basal and modulated intracellular signaling activity in multiple cell subpopulations. Previously, SCNP analysis of a broad panel of immune signaling pathways in cell subsets within PBMCs from 60 healthy donors identified a race-associated difference in B cell anti-IgD-induced PI3K pathway activity.

**Methods:**

The present study extended this analysis to a broader range of signaling pathway components downstream of the B cell receptor (BCR) in European Americans and African Americans using a subset of donors from the previously analyzed cohort of 60 healthy donors. Seven BCR signaling nodes (a node is defined as a paired modulator and intracellular readout) were measured at multiple time points by SCNP in PBMCs from 10 healthy donors [5 African Americans (36-51 yrs), 5 European Americans (36-56 yrs), all males].

**Results:**

Analysis of BCR signaling activity in European American and African American PBMC samples revealed that, compared to the European American donors, B cells from African Americans had lower anti-IgD induced phosphorylation of multiple BCR pathway components, including the membrane proximal proteins Syk and SFK as well as proteins in the PI3K pathway (S6 and Akt), the MAPK pathways (Erk and p38), and the NF-κB pathway (NF-κB). In addition to differences in the magnitude of anti-IgD-induced pathway activation, racial differences in BCR signaling kinetic profiles were observed. Further, the frequency of IgD+ B cells differed by race and strongly correlated with BCR pathway activation. Thus, the race-related difference in BCR pathway activation appears to be attributable at least in part to a race-associated difference in IgD+ B cell frequencies.

**Conclusions:**

SCNP analysis enabled the identification of statistically significant race-associated differences in BCR pathway activation within PBMC samples from healthy donors. Understanding race-associated contrasts in immune cell signaling responses may be one critical component for elucidation of differences in immune-mediated disease prevalence and treatment responses.

## Background

Racial differences have been documented in the prevalence of autoimmune diseases such as systemic lupus erythematosus [1] and multiple sclerosis [2] and in the clinical response to immunotherapies [such as IFN-α [3] and Benlysta/belimumab [4]]. However, the biologic basis for such race-associated differences remains poorly understood. A better understanding of the underlying biologic mechanisms of race-associated differences in immune signaling responses may provide clinically relevant information regarding the mechanisms underlying race-related differences in treatment responsiveness.

Single-cell network profiling (SCNP) is a multiparametric flow cytometry-based approach that enables the simultaneous measurement of basal and evoked signaling in multiple cell subpopulations [5]. Recently, SCNP technology was applied to quantify immune signaling pathway activation following modulation with 12 immunomodulators (including IFN-α, IFN-γ, IL-2, IL-4, IL-6, IL-10, IL-27, anti-IgD, LPS, R848, PMA, and CD40L) in 7 distinct immune cell subpopulations within PBMC samples from 60 healthy donors [6]. Using a training/test set approach, race-associated differences in anti-IgD-induced levels of p-S6 and p-Akt in B cells were identified [6]. The present study was performed to analyze anti-IgD-induced modulation of a broader range of BCR signaling pathway components at multiple time points using a subset of European American (EA) and African American (AA) donor samples from the previously analyzed healthy donor cohort [7].

## Methods

### PBMC samples

Cryopreserved PBMC samples collected from 10 healthy donors [5 AAs (mean age 45.4 yrs), 5 EAs (mean age 48.6 yrs), all males (Table1)] within the Department of Transfusion Medicine, Clinical Center, National Institutes of Health with Institutional Review Board approval were used in this study. All blood samples, donated for research purposes with informed consent, were collected and processed as described previously [8].

**Table 1:** Summary of donor numbers, age, race, and gender

	**AA**	**EA**
No. of Donors	5	5
Mean Age (y)	45.4	48.6
Age Range (y)	36-51	36-56
Gender	5 Male	5 Male

### SCNP assay

Cryopreserved PBMC samples were thawed at 37°C and resuspended in RPMI 1640 (1% FBS) before staining with amine aqua viability dye (Invitrogen, Carlsbad, CA). Cells were resuspended in RPMI 1640 (10% FBS), aliquoted to 100,000 cells per well in 96-well plates, and rested for 2 h at 37°C prior to incubation with anti-IgD 5 μg/ml (BD, San Jose, CA) or anti-IgM 10 μg/ml (Southern Biotech, Birmingham, AL). After modulation with anti-IgD (for 5′, 15′, 30′, or 60′) or anti-IgM (5′), cells were fixed with paraformaldehyde and permeabilized with 100% ice-cold methanol as previously described [9]. Permeabilized cells were washed with FACS buffer (PBS, 0.5% BSA, 0.05% NaN_3_), pelleted, and stained with fluorochrome-conjugated Abs. Abs used include anti-CD20 (clone H1), -p-NF-κB (clone K10-895.12.50), -c-poly(ADP-ribose) polymerase (clone F21-852), -p-Erk (clone 20A), -p-SFK/Lck (clone 4/LckY505), -p-p38 (clone 36/p38), -p-Syk (clone 17a/P-ZAP70) [BD, San Jose CA]; -p-Akt (clone D9E), -p-S6 (clone 2F9) [CST, Danvers, MA]; and -IgD [Southern Biotech, Birmingham, AL].

### Flow cytometry data acquisition and analysis

**Figure 1:**
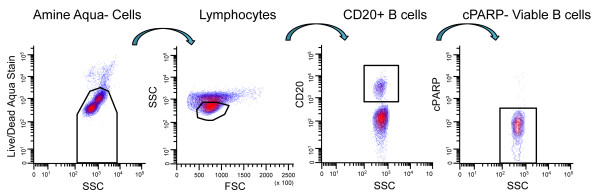
**Gating strategy used to delineate the viable B cell subpopulation within PBMCs.** Boolean logic was used to identify cells that were amine aqua negative, lymphocytes by light scatter, positive for CD20, and negative for c-poly(ADP-ribose) polymerase (c-PARP).

Flow cytometry data was acquired using FACS DIVA software (BD, San Jose, CA) on an LSRII Flow Cytometer (BD, San Jose, CA). All flow cytometry data were analyzed with WinList (Verity House Software, Topsham, ME). For all analyses, dead cells and debris were excluded by forward scatter (FSC), side scatter (SSC), and amine aqua viability dye. Viable B cells were delineated according to the gating scheme shown in Figure1.

### SCNP terminology and metrics

The term “signaling node” refers to a specific protein readout in the presence or absence of a specific modulator. For example, the response to anti-IgD stimulation can be measured using p-S6 as a readout. This signaling node is designated “anti-IgD → p-S6”. The “Fold” metric was applied to measure the level of anti-IgD-induced modulation of each signaling molecule after modulation compared to its level in the basal state. The “Fold” metric was calculated as follows:

(1) $$\text{Fold}:\left({\text{log}}_{2}\left[\text{MFI}\left(\text{Modulated}\right)/\text{MFI}\left(\text{Unmodulated}\right)\right]\right)$$

Where MFI is the median fluorescence intensity.

### Statistical analyses

Statistical differences between groups were assessed by the Wilcoxon rank sum test. For each of the 7 phospho-protein readouts, race-associated differences were assessed by averaging the Fold values over the 4 modulated time points (5’, 15’, 30’, 60’) and applying the Wilcoxon rank sum test to the averaged Fold value. One donor was excluded from all analyses due to an insufficient number (<200) of B cells (“events”) collected by the flow cytometer.

### Correlations between signaling nodes

R software (version 2.12.1) was used to compute Pearson correlation coefficients between IgD+ B cell frequency and BCR signaling nodes. Heatmaps were generated in Excel 2007 (Microsoft, Redmond, WA).

## Results and discussion

Seven signaling nodes (anti-IgD → p-S6, anti-IgD → p-Syk, anti-IgD → p-Akt, anti-IgD → p-Erk, anti-IgD → p-SFK, anti-IgD → p-p38, and anti-IgD → p-NF-κB), or specific protein readouts of modulated signaling, were measured in viable B cells (defined as amine aqua negative, forward scatter/side scatter low, CD20+, c-poly(ADP-ribose) polymerase (c-PARP) negative cells, Figure1) at several time points following modulation with anti-IgD in PBMCs collected from 10 healthy donors (Table1). The “Fold” metric (see Materials and Methods) was applied to measure the level of a signaling molecule after modulation compared to its level in the basal state.

Averaged over all donors, viable B cells constituted 18.3% (standard deviation = 8.8%, range = 2.2–28.1%) of the parental Lymphocyte subpopulation (see Figure1). For one donor, the CD20+ B cell percentage was extremely low (2.2% of Lymphocytes) resulting in too few B cells for analysis (i.e. <200 B cells in most wells of the 96-well plate). Therefore, data for this donor was excluded from the analysis presented below.

### Race-associated differences in BCR signaling pathway activation

**Figure 2:**
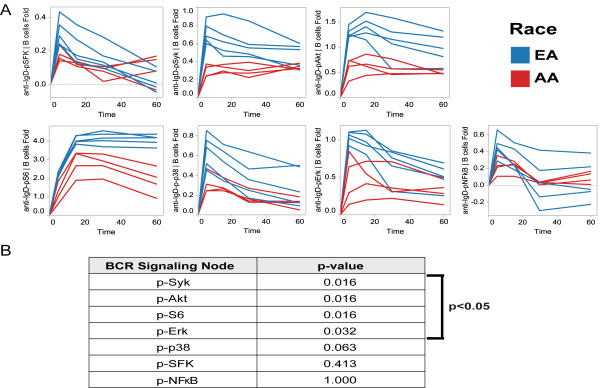
**BCR signaling activation in AA and EA donors.***A*, Fold vs. time is shown for the 7 phospho-protein readouts for 9 of the 10 healthy donors (one AA donor was excluded due to an insufficient number of B cells (<200 events) collected for analysis). *B*, Race-associated differences were assessed by the Wilcoxon rank sum test (see Materials and Methods). Four phospho-protein readouts had a statistically significant race-associated difference (p < 0.05).

On average, compared with B cells from EA donors, B cells from AA donors displayed lower anti-IgD-induced phosphorylation for all 7 of the tested BCR pathway components (Figure2A). For the p-Syk, p-S6, p-Akt, and p-Erk signaling readouts, the race-associated difference in the magnitude of anti-IgD-induced activation levels (quantified by the Fold and averaged over all time points, see Materials and Methods) reached statistical significance (p < 0.05, Wilcoxon test, Figure2B). To assess whether or not the race-associated difference in BCR pathway activation is specific to modulation with anti-IgD, the 7 phospho-proteins were measured following modulation with anti-IgM (5′) and higher induced phosphorylation was observed in EA donors than in AA donors, reaching statistical significance (p < 0.05, Wilcoxon test) for p-Syk, p-S6, p-Akt, and p-SFK (data not shown). Future studies will be performed in larger independent donor cohorts with greater statistical power to assess the external validity of the race-associated differences observed in this exploratory study.

### BCR signaling kinetics in AA and EA donors

For the BCR signaling pathway components p-SFK, p-p38, and p-NF-κB, a rapid, transient increase in signal was observed, peaking at the 5 minute time point followed by a steady decrease until the 60 minute time point. For these three BCR signaling components, the kinetic profiles were comparable between AA and EA donors. Previous studies have reported similar kinetic profiles for p-p38 in human CD20+ B cells following BCR cross-linking with anti-IgM and anti-IgG [10].

In contrast, anti-IgD-induced signaling kinetics for p-Syk, p-Akt, and p-S6 appeared different among EA and AA donors (Figure2A). Specifically, increases for both p-Syk and p-Akt were at or near peak values at 5 minutes and, in EA donors, gradually declined through the 60 minute time point [with an average difference in the Fold between the 5′and 60′ time point of 0.25 (for p-Syk) and 0.32 (for p-Akt)], while peak levels (although lower in magnitude) were generally sustained through the 60 minute time point in AA donors [with an average difference in the Fold between the 5’ and 60’ time point of −0.04 (for p-Syk) and 0.11 (for p-Akt)]. In recent analyses using B cells from healthy donors, anti-IgM/anti-IgG-induced Syk phosphorylation was reported to peak between 4 and 8 minutes and return to basal levels by 90 minutes [10] thus displaying a signaling kinetic profile similar to the p-Syk profile observed here for EA donors.

For anti-IgD-induced p-S6, peak activity was observed at 15 minutes (i.e. delayed relative to more upstream pathway components p-Akt and p-Syk) and, in EA donors, the p-S6 levels were sustained through the 60 minute time point (with an average difference in the Fold between the 15’ and 60’ time point of 0.02), while p-S6 levels had declined by the 60 minute time point in AA donors (with an average difference in the Fold between the 15’ and 60’ time point of 0.98).

To assess BCR signaling responses at a network level, Pearson correlation coefficients between pairs of signaling nodes at each time point were calculated. All of the responses at the 5’ time point were positively correlated (r > 0.6), and positive correlations were observed between the majority of the nodes at later time points (i.e. 15’, 30’, and 60’) with the exception of the node-to-node correlations with p-NF-κB or p-SFK, nodes which displayed low or no induced activation (average Folds < 0.3) at later time points (Figure2A).

### Race-associated differences in IgD + B cell percentages

Given that race-associated differences in anti-IgD responses were observed in the overall viable B cell population, it is of interest to determine if the race-associated difference in anti-IgD responses is due to a race-associated difference in anti-IgD responses specifically within the IgD+ B cell subpopulation and/or due to a race-associated difference in the frequency of IgD+ B cells. Although it would be ideal to measure anti-IgD responses directly within IgD+ B cells, modulation with anti-IgD can interfere with the subsequent detection of surface IgD with fluorescently-labeled anti-IgD. Therefore, an indirect approach was taken whereby the IgD+ B cell frequency was determined in the unmodulated condition and the relationship between IgD+ B cell frequencies and anti-IgD responses within the overall viable B cell population was assessed.

**Figure 3:**
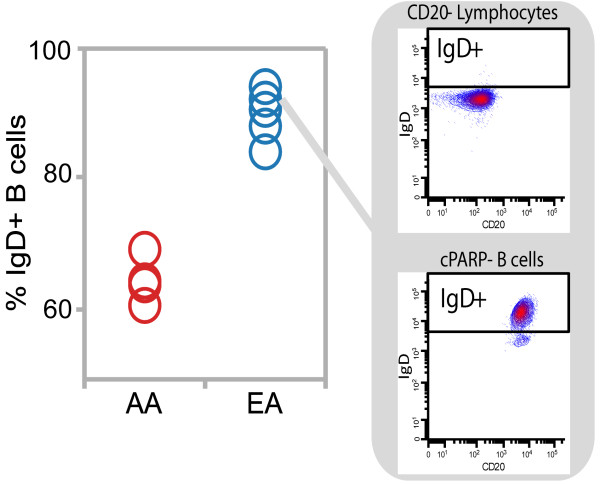
**IgD+ B cell percentages in AA and EA donors.** CD20- lymphocytes were used as an internal negative control to define the IgD+ region for each donor. The IgD+ region was applied to the viable B cell subpopulation to determine the IgD+ B cell percentage for each donor. Representative flow plots from one healthy donor are shown. IgD+ B cell percentages were assessed under the unmodulated condition. EAs had a higher percentage of IgD+ B cells than AAs and the race-associated difference was significant (p = 0.016, Wilcoxon test).

The percentage of IgD+ B cells was determined for each donor by using the CD20- Lymphocyte subpopulation within each donor PBMC sample as an internal negative control to define an IgD+ region which was subsequently applied to the viable B cell subpopulation (Figure3). The percentage of IgD+ B cells was significantly lower in AA donor samples than in EA donor samples (p = 0.016, Wilcoxon test). Despite a significant race-associated difference in the percentage of IgD+ B cells, the level of IgD expression within the IgD+ B cell subset (as quantified by the median fluorescence intensity (MFI) of IgD in the IgD+ B cell subset) did not differ between the races (p = 0.286, Wilcoxon test).

**Figure 4:**
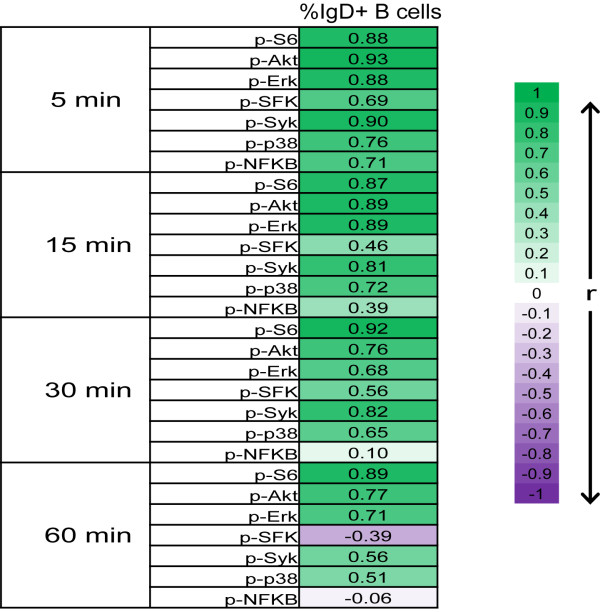
**Correlations between IgD+ B cell percentages and BCR signaling pathway activation.** The heatmap shows the magnitude of the Pearson correlation coefficients (r) between the IgD+ B cell percentage and the Fold for each signaling node at each anti-IgD modulation time point.

The correlation between IgD+ B cell frequency and BCR signaling pathway activation was then assessed by calculating the Pearson correlation coefficient, r, between IgD+ B cell percentage and the Fold metric for each BCR signaling node at each anti-IgD modulation time point (Figure4). A positive correlation (defined as r > 0.6) was observed for the majority of BCR signaling responses measured, with the exception of p-NF-κB and p-SFK at the 15’, 30’, and 60’, [low or no induced activation (average Folds < 0.3) was observed for these nodes at these time points (see Figure2A)].

**Figure 5:**
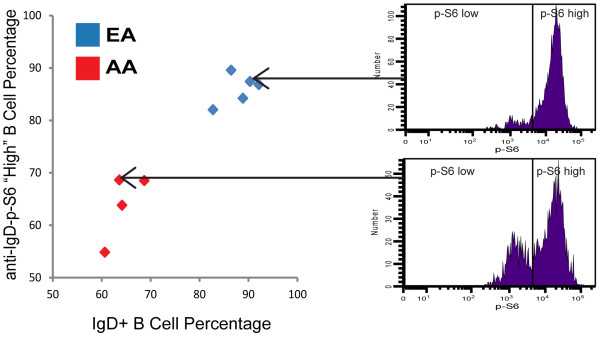
**IgD+ B cell percentage vs. percentage of anti-IgD responsive (p-S6 “high”) B cells**. Anti-IgD-induced p-S6 histograms are shown for 2 representative donors. p-S6 “high” and p-S6 “low” regions were established by estimating the location of the trough in the bimodal profile for one donor and the same regions were applied to all donors. The percentage of anti-IgD induced p-S6 “high” B cells had a statistically significant race-associated difference (p = 0.016, Wilcoxon test). The ratio of the MFI in the p-S6 “high” region to the MFI in the p-S6 “low” region did not differ between the races (p = 0.905, Wilcoxon test).

For the anti-IgD induced p-S6 response at the 15’ time point, pronounced bimodality was observed allowing for a quantitative assessment of the percentage of p-S6 “high” (i.e. anti-IgD responsive B cells). The percentage of anti-IgD responsive B cells closely approximated the percentage of IgD+ B cells for each donor (Figure5). While the percentage of p-S6 “high” cells was significantly different between the two racial groups (p = 0.016, Wilcoxon test), the ratio of the MFI in the p-S6 “high” region to the MFI in the p-S6 “low” region did not differ between the races (p = 0.905, Wilcoxon test).

Overall, these results suggest that the observed race-associated difference in BCR signaling pathway activation could be attributed at least in part to a race-associated difference in IgD+ B cell frequencies (and thus the percentage of B cells that respond to anti-IgD) and not to surface levels of IgD or intrinsic differences in the magnitude of the anti-IgD induced response within each B cell.

Within the peripheral blood, the IgD+ B cell subpopulation consists of naïve (CD27-) B cells and non-switched (IgE-/IgG-/IgA-) memory (CD27+) B cells, while the IgD- B cell subpopulation consists primarily of switched (IgE+/IgG+/IgA+) memory (CD27+) B cells [11,12]. Thus, in the donor set studied here, AAs may have a greater frequency of switched memory B cells than EA donors. Studies which include additional B cell phenotypic markers, such as CD27 and IgM, are currently underway to assess race-related differences in more well-defined B cell subsets. To our knowledge, no prior studies have reported race-associated differences in B cell subset frequencies. However, changes in the composition of the peripheral B cell pool have been reported for systemic lupus erythematosus [13], common variable immunodeficiency [14], and with age [15].

Establishing the range of BCR signaling responses in healthy donors can enable the identification of dysregulated signaling in disease states by providing a reference range to which signaling responses from diseased samples can be compared. Recently, BCR signaling profiles from follicular lymphoma B cells were compared with signaling profiles from B cells from healthy blood donors to identify alterations in BCR-mediated signaling in follicular lymphoma, and BCR-mediated phosphorylation of p38 was found to be lower in follicular lymphoma than in a reference healthy blood donor [16]. The data presented here demonstrating that BCR-mediated signaling within healthy donors can vary with at least one demographic variable (race) highlights the importance of using healthy donor cohorts consisting of individuals matched to the diseased population by demographic characteristics such as age, race, and gender. Such criteria may be critical in establishing reference signaling ranges as a basis for comparison with diseased signaling responses.

## Conclusions

In this study, differences in B cell subset frequencies and BCR-mediated signaling pathway activation were found amongst AA and EA donors. Subsequent studies will extend the SCNP approach used here to assess differences in B cell subpopulation frequencies and BCR signaling in additional ethnic groups. Controlling for ethnicity is emerging as a key component in assuring the accuracy of clinical diagnostics [17] and in selecting treatments [4]. Ultimately, a greater understanding of race-associated differences in immune cell signaling responses may help to elucidate the basis for racial differences in immune-mediated disease prevalence and treatment response rates.

## Abbreviations

AA: African American; EA: European American; SCNP: Single-cell network profiling.

## Competing interests

DML, BL, REH, and AC are employees of Nodality Inc. KM is an employee of MedImmune. The other authors declare no financial conflicts of interest.

## Authors’ contributions

DML, ZP, EW, REH, KM, FMM, and AC conceived the study. DML and KM performed the experiments. DML and BL analyzed the experiments and drafted the manuscript. All authors read and approved the final manuscript.
